# 583. *Candida Auris* Outbreak in the Burn Unit: Risk Factors and Characteristics

**DOI:** 10.1093/jbcr/irag033.356

**Published:** 2026-04-06

**Authors:** Nicholas C Gorman, Melanie Wellington, Alexander Kurjatko, Nicole Wiltfang, Kristin L Varzavand, Colette Galet, Samuel W Jones, Karen Brust

**Affiliations:** University of Iowa, Carver College of Medicine, Iowa City, IA; University of Iowa Health Care, Iowa City, IA; University of Iowa, Iowa City, IA; University of Iowa Health Care, Iowa City, IA; University of Iowa Health Care, Iowa City, IA; University of Iowa, Iowa City, IA; University of Iowa Health Care, Iowa City, IA; University of Iowa Health Care, Iowa City, IA

## Abstract

**Introduction:**

Candida auris (*Corynebacterium auris*) is an emerging multidrug-resistant fungal pathogen increasingly recognized as a cause of nosocomial outbreaks. Burn patients are at particular risk of acquiring this pathogen because of impaired skin integrity, frequent invasive procedures, immune dysfunction, and treatment with broad-spectrum antimicrobials. Herein, we describe our experience with *C. auris* in a 17-bed burn unit, to better understand their colonization and infection risks.

**Methods:**

This is a program improvement project. Our epidemiology team collected information on burn patients who tested positive for *C. auris* from 10/29/2024 to 6/30/2025. Demographics, comorbidities, hyperglycemia on admission, burn injury information and management, and *C. auris* detection information were collected. Descriptive statistics were obtained.

**Results:**

Eight burn patients were positive for *C. auris*, five (62.5%) via polymerase chain reaction as a part of point prevalence studies and three (37.5%) by culture. Six (75%) were defined as *C. auris* colonization and two (25%) as *C. auris* infections based on receipt of targeted therapy. All patients were placed on broad spectrum antibiotics. The median time from admission to positivity was 17 days [IQR: 10-30.8]. All patients were white and male with a median age of 55 years [IQR: 32-74]. Only two presented with a history of diabetes mellitus, one had a history of neoplastic disease. None had a history of chronic kidney disease, immunocompromised state, or recent surgery. The median total burn surface area (TBSA) was 17% [IQR: 5.6-48.8]. Six (75%) presented full thickness burns and three (37.5%) had inhalation injury. All patients were hyperglycemic on admission (glucose median level:174 [IQR: 144-199]). Four (50%) had a central venous line, seven (87.5%) had an indwelling urinary catheter. Four (50%) required mechanical ventilation and stayed on a ventilator for an average of 15 days. The median hospital length of stay was 47 days [IQR: 13-51].

**Conclusions:**

Over a 9-month period, eight burn patients were positive for *C. auris* colonization and infection. A positive *C. auris* culture led to screening procedures to identify transmission on the unit to other patients. Some traditional risk factors were not met for acquisition of *C. auris*, such as time in other facilities. Multiple traditional risk factors were present, including use of medical devices, prolonged inpatient stays, frequent surgical procedures, and widespread use of antimicrobials.

**Applicability of Research to Practice:**

Burn units should consider a *C. auris* surveillance program for patients in a burn unit should *C. auris* be identified in a clinical specimen. Specifically, facilities may consider routine surveillance for patients with prolonged stays and/or risk factors. Further study is warranted to identify the clinical significance of *C. auris* positivity in burn-injured populations.

**Funding for the study:**

N/A.

